# Gene Set Based Integrated Methylome and Transcriptome Analysis Reveals Potential Molecular Mechanisms Linking Cigarette Smoking and Related Diseases

**DOI:** 10.1089/omi.2023.0028

**Published:** 2023-05-17

**Authors:** Pashupati P. Mishra, Binisha H. Mishra, Emma Raitoharju, Nina Mononen, Jorma Viikari, Markus Juonala, Nina Hutri-Kähönen, Mika Kähönen, Olli T. Raitakari, Terho Lehtimäki

**Affiliations:** ^1^ Department of Clinical Chemistry, Faculty of Medicine and Health Technology, Tampere University, Tampere, Finland.; ^2^ Finnish Cardiovascular Research Center Tampere, Faculty of Medicine and Health Technology, Tampere University, Tampere, Finland.; ^3^ Department of Clinical Chemistry, Fimlab Laboratories, Tampere, Finland.; ^4^ Molecular Epidemiology, Faculty of Medicine and Health Technology, Tampere University, Tampere, Finland.; ^5^ Tampere University Hospital, Tampere, Finland.; ^6^ Department of Medicine, University of Turku, Turku, Finland.; ^7^ Division of Medicine, Turku University Hospital, Turku, Finland.; ^8^ Department of Paediatrics, Tampere University Hospital, Faculty of Medicine and Health Technology, Tampere University, Tampere, Finland.; ^9^ Department of Clinical Physiology, Tampere University Hospital, Tampere, Finland.; ^10^ Research Centre of Applied and Preventive Cardiovascular Medicine, University of Turku, Turku, Finland.; ^11^ Department of Clinical Physiology and Nuclear Medicine, Turku University Hospital, Turku, Finland.; ^12^ Centre for Population Health Research, University of Turku and Turku University Hospital, Turku, Finland.

**Keywords:** cigarette smoking, gene set analysis, DNA methylation, transcriptomics, public health, epigenomics

## Abstract

Advanced integrative analysis of DNA methylation and transcriptomics data may provide deeper insights into smoke-induced epigenetic alterations, their effects on gene expression and related biological processes, linking cigarette smoking and related diseases. We hypothesize that accumulation of DNA methylation changes in CpG sites across genomic locations of different genes might have biological significance. We tested the hypothesis by performing gene set based integrative analysis of blood DNA methylation and transcriptomics data to identify potential transcriptomic consequences of smoking via changes in DNA methylation in the Young Finns Study (YFS) participants (*n* = 1114, aged 34–49 years, women: 54%, men: 46%). First, we performed epigenome-wide association study (EWAS) of smoking. We then defined sets of genes based on DNA methylation status within their genomic regions, for example, sets of genes containing hyper- or hypomethylated CpG sites in their body or promoter regions. Gene set analysis was performed using transcriptomics data from the same participants. Two sets of genes, one containing 49 genes with hypomethylated CpG sites in their body region and the other containing 33 genes with hypomethylated CpG sites in their promoter region, were differentially expressed among the smokers. Genes in the two gene sets are involved in bone formation, metal ion transport, cell death, peptidyl-serine phosphorylation, and cerebral cortex development process, revealing epigenetic–transcriptomic pathways to smoking-related diseases such as osteoporosis, atherosclerosis, and cognitive impairment. These findings contribute to a deeper understanding of the pathophysiology of smoking-related diseases and may provide potential therapeutic targets.

## Introduction

Cigarette smoking, referred to as smoking hereafter, is the leading preventable risk factor for numerous diseases and affects almost every organ in the body such as heart, blood vessels, lungs, brain, liver, mouth, and bones (Bergen, 1999). Smoking induces diseases through pathogenic mechanisms such as inflammation, oxidative damage, endothelial dysfunction, and alterations in the immune system and epigenetics (Zhou et al., 2016).

However, the molecular cascade underlying these mechanisms is not well understood. Availability of high-throughput multi-omics data and advanced statistical techniques for integrative analysis makes it possible to investigate the interrelationships of the involved molecules and their functions in a biological system across multiple molecular layers.

Understanding the underlying molecular mechanism of smoking-related disease induction is crucial for the development of interventions, treatments, and pharmacological agents aimed at reducing smoking-related health burden. For instance, knowledge of the epigenetic and consequent transcriptomic effects of smoking may aid in the development of drugs targeting specific epigenetic mechanisms to prevent consequences at transcriptomic level.

Such an approach is already in use for cancer treatment (Tzika et al., 2018). The drugs, when used along with the existing evidence-based therapies for treating smoking (Fiore et al., 2000), may play a crucial role in smoking-related health risks management.

Several studies have shown smoking-related alterations in DNA methylation (Joehanes et al., 2016; Kaur et al., 2019; Mishra et al., 2020; Zeilinger et al., 2013). Smoking can affect DNA methylation via several mechanisms. For example, carcinogenic contents of cigarette smoke such as arsenic, chromium, formaldehyde, polycyclic aromatic hydrocarbons, and nitrosamines can damage DNA. The DNA damage leads to recruitment of DNA methyltransferase 1 (DNMT1) at the repair site, consequently affecting DNA methylation (Lee and Pausova, 2013). Also, nicotine in cigarette smoke can affect DNA methylation by directly downregulating DNMT1 expression.

Cigarette smoke may also alter DNA methylation indirectly through the modulation of expression and activity of DNA-binding factors. The largest epigenome-wide association study (EWAS) of smoking identified 18,760 active smoking-related CpG sites annotated to 7201 genes with false discovery rate (FDR) <0.05 (Joehanes et al., 2016).

This study was based on the Infinium HumanMethylation 450 K BeadChip that measures methylation at about 450,000 CpG sites throughout the genome instead of the >850,000 CpG sites measured by the newer Illumina HumanMethylationEPIC BeadChip (EPIC). A recent EWAS of smoking habit using EPIC chip identified 952 CpG sites in 500 genes differentially methylated between current and never smokers at a genome-wide significance threshold (*p* = 6.25 × 10^−8^) (Christiansen et al., 2021).

The impact of smoking on DNA methylation (Joehanes et al., 2016; Kaur et al., 2019; Mishra et al., 2020; Zeilinger et al., 2013) and genome-wide expression (Charlesworth et al., 2010; Huan et al., 2016; Vink et al., 2017) have previously been studied mainly independently. Integrative studies on consequences of the altered DNA methylation on genome-wide expression on the same set of individuals in blood have been based on 450 K chip (Maas et al., 2020; Tsai et al., 2018).

A few multi-omic integrative studies on smoking are based on EPIC chip-based DNA methylation data; however, they are based on specific cell types such as bronchoalveolar lavage cells (Ringh et al., 2019) and monocytes (Wan et al., 2018) but not on whole blood. While the earlier mentioned integrative multi-omic studies on smoking have crucial contributions to understanding of smoking-related disease mechanisms, integrative analysis of DNA methylome and transcriptome from whole blood using EPIC array is an important scientific gap that needs to be addressed.

Further, the previous integrative analyses were based on traditional one-to-one association analysis between genes and methylation sites, which is likely to suffer from lack of sufficient statistical power.

Small changes in individual gene expression levels associated with smoking-related methylation pattern in their genomic regions can be missed by one-to-one association analysis of methylation sites and corresponding genes due to lack of statistical power. Combining genes with smoking specific CpG sites and analyzing them as a set increases the probability of finding transcriptomic consequence of smoking via epigenetic route due to increased statistical power.

Small but coordinated changes in gene expression due to smoking-related DNA methylation changes in their genomic regions can have major biological effects even if the changes are not statistically significant for individual genes. Gene set analysis can capture such results as we have shown in our previous study using 450 K chip-based methylation data analysis (Mishra et al., 2020).

**FIG. 1.**
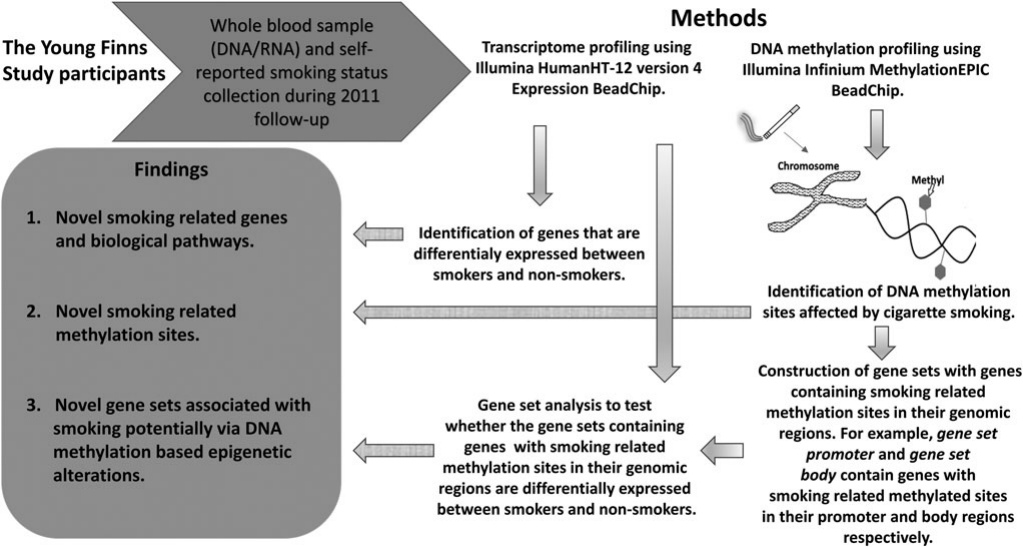
Schematic representation of the overall study design.

In the present study, we performed advanced gene set based integrative analysis of DNA methylome with 850 K methylation sites and transcriptome from whole blood collected from a cohort of European ancestry to test whether sets of genes containing smoking specific CpG sites within their genomic region (cis-regulation) are differently expressed among smokers, as compared with non-smokers (Fig. 1). In addition to the gene set based integrative analysis, we also conducted individual omics data analysis for the DNA methylation and transcriptome data to identify smoking-related CpG sites and genes respectively (Fig. 1).

## Materials and Methods

### Cohort description

This study was based on the Young Finns Study (YFS), one of the largest existing prospective multicenter follow-up studies assessing cardiovascular risk factors from childhood to adulthood (Raitakari et al., 2008). The study began in 1980 with 3596 children and adolescents aged 3–18 years randomly selected from 5 university hospital areas in Finland (Turku, Tampere, Helsinki, Kuopio, and Oulu) and they have been followed in regular intervals for over 40 years until 2020.

The study was approved by the Ethics Committee of the Hospital District of Southwest Finland on June 20, 2017 (ETMK: 68/1801/2017). All participants gave their written informed consent, and the studies were conducted in accordance with the Declaration of Helsinki. Data protection will be handled according to current regulations as noted next.

This study involved a subpopulation of 1114 participants from the YFS with DNA methylation, transcriptomics, smoking habit, alcohol consumption, and occupation-based socio-economic status data available. Variable for smoking habit was based on self-reported information on whether the participants are daily smokers or never or less than daily smokers. Variable for the participants' alcohol consumption information was generated from their self-reports on their alcohol consumption during the previous week where 1 unit is equivalent to 14 g of alcohol (Juonala et al., 2009).

### DNA methylation profiling, pre-processing, and normalization

DNA was obtained from EDTA-blood samples collected during the YFS 2011 follow-up using a Wizard^®^ Genomic DNA Purification Kit (Promega Corporation, Madison, WI, USA) according to the manufacturer's instructions. DNA integrity was tested by analyzing a subset of the samples with Agilent's Fragment Analyzer. Genome-wide DNA methylation levels were obtained using Illumina Infinium MethylationEPIC BeadChips, following the protocol by Illumina (Marttila et al., 2021). All pre-processing steps were performed using functions implemented in the *minfi* R/Bioconductor package (Fortin et al., 2017).

All analyzed samples have a sum of detection *p*-values <0.01 across all the probes. Only the samples with logged (log2) median of the methylated and unmethylated intensities clustering well based on default threshold of *plotQC* function in *minfi* R package were included. Samples for which the actual sex did not match the predicted sex were excluded.

Background subtraction and dye-bias normalization were performed via the noob method (Triche et al., 2013), followed by stratified quantile normalization using *preprocessQuantile* function in *minfi*. Probes with a detection *p*-value of more than 0.01 in more than 99% of the samples were filtered out.

CpG loci on sex chromosomes were excluded from the analysis to avoid gender-based methylation bias. Also, cross-reactive probes (McCartney et al., 2016; Pidsley et al., 2016) and probes with single nucleotide polymorphisms were excluded from the analysis. After quality control, the total number of autosomal CpGs was 769,683 in 1114 samples (150 active smokers and 964 never or less than daily-smokers).

### Transcriptome profiling pre-processing and normalization

RNA isolation was performed from whole-blood samples collected from the YFS participants during the 2011 follow-up. Expression levels were analyzed with Illumina HumanHT-12 version 4 Expression BeadChip (Illumina, Inc.), containing 47,231 expression and 770 control probes. Raw Illumina summary probe-level data were exported from Beadstudio and processed in R (www.r-project.org) using a nonparametric background correction, followed by quantile normalization with control and expression probes, with the *neqc* function in the *limma* package (Smyth et al., 2005) and a log2 transformation. The pre-processing details are also described elsewhere (Mishra et al., 2021).

### Biostatistical analysis

All statistical analyses were performed using R statistical software (v.4.1.0) (R Core Team, 2021).

### Availability of data and materials

The dataset supporting the conclusions of this article was obtained from the Cardiovascular Risk in YFS, which comprises health-related participant data. The use of data is restricted under the regulations on professional secrecy (Act on the Openness of Government Activities, 612/1999) and on sensitive personal data (Personal Data Act, 523/1999, implementing the EU data protection directive 95/46/EC). Due to these restrictions, the data cannot be stored in public repositories or otherwise made publicly available.

Data access may be permitted on a case-by-case basis on request only. Data sharing outside the group is done in collaboration with the YFS group and requires a data-sharing agreement. Investigators can submit an expression of interest to the chairperson of the publication committee, Prof Mika Kähönen (Tampere University, Tampere, Finland) and Prof Terho Lehtimäki (Tampere University).

#### DNA methylation data analysis

Beta values, calculated as the ratio of intensities between methylated and unmethylated probe, were used as measures of methylation level. Differentially methylated positions (DMPs) for smoking status were identified using moderated *t*-test implemented in *limma* package in R (Smyth et al., 2005). The analysis was adjusted for age, sex, body mass index (BMI), alcohol consumption, socioeconomic status, and blood cell type proportions by adding them as covariates in the linear model implemented in *limma*.

Blood cell type proportions consisted of proportions of CD8T, CD4T, natural killer cells, B cells, monocytes, and granulocytes in white blood cells estimated through the reference-based Houseman method (Houseman et al., 2012) using the estimateCellCounts function in the minfi Bioconductor package in R (Aryee et al., 2014). Population structure, batch effects, and technical covariates were corrected for by including the first 30 principal components based on both control probes and methylation beta values each as covariates in the multiple linear regression model (Lehne et al., 2015).

Physical activity index, measured as weekly metabolic equivalent task hours (MET-h/week) calculated from information on the frequency, intensity, and duration of physical activity including leisure-time physical activity and commuting to the workplace (Pälve et al., 2018), was significantly associated with smoking habit with odds ratio (OR) of 0.88 and *p*-value of 2.8 × 10^−09^. Alcohol consumption was also significantly associated with smoking habit but with less statistical significance (OR = 1.13, *p* = 1.9 × 10^−05^) as compared with that of the physical activity index.

Therefore, as our main objective was to accurately estimate the individual effect of smoking habit on DNA methylation, we did not adjust the analysis with physical activity index to avoid the collinearity-related problem. Genomic control to reduce the number of false positive results was done by calculating genomic inflation factor (λ) as the ratio of the median of the empirically observed distribution of the test statistic to the expected median.

Novelty of the identified DMPs was assessed through a literature review, including a comparison of the results with the largest (Joehanes et al., 2016) and the latest (Christiansen et al., 2021) studies at FDR <0.05. Differentially methylated CpGs with genome-wide significance of *p* < 6.5 × 10^−8^ (0.05/769,683) that are annotated to genes were used to define sets of genes.

A total of 16 gene sets were defined with genes containing hypo- or hypermethylated CpG sites in their: (1) transcription start site (TSS) 1500 region (200–1500 bases upstream of the TSS), (2) TSS200 region (0–200 bases upstream of the transcriptional start site), (3) 5′UTR region (within the 5′ untranslated region, between the TSS and the ATG start site), (4) first exon region (first exon of the gene), (5) body region (between the ATG and stop codon irrespective of the presence of introns, exons, TSS, or promoters), (6) 3′UTR region (between the stop codon and poly A signal), (7) exon boundary region, and (8) promoter region (TSS200, TSS1500, 5′UTR, and first exon regions combined).

Definitions of the gene regions were based on Human Methylation 850 K array and were obtained from *Illumina Human Methylation EPICanno. ilm10b2. hg19* R/Bioconductor package (Hansen et al., 2016). Differentially expressed genes (DEGs) among the smokers identified with transcriptome data analysis in this study (Section transcriptome data analysis) as well as in previous studies were removed from the defined gene sets because the aim of gene set analysis in this study was to test whether the method can identify transcriptomic consequences of smoking-related alterations in DNA methylation that is missed by traditional univariate methods.

#### Transcriptome data analysis

The DEGs between smokers and non-smokers were identified using moderated *t*-test implemented in *limma* R package. The analysis was adjusted for age, sex, BMI, alcohol usage, socioeconomic status, and the first 10 principal components of the transcriptomic data. Enrichment analysis of the statistically significant DEGs with FDR <0.05 with biological process terms of gene ontology (GO) database (Ashburner et al., 2000; Carbon et al., 2021) was done using *clusterProfiler* R package (Wu et al., 2021; Yu et al., 2012).

The enrichment analysis was done for up- and downregulated genes separately. Summarization and interpretation of the biological process GO terms was done using REVIGO using default parameters (Supek et al., 2011).

#### Integrative DNA methylation and transcriptomic data analysis

**FIG. 2.**
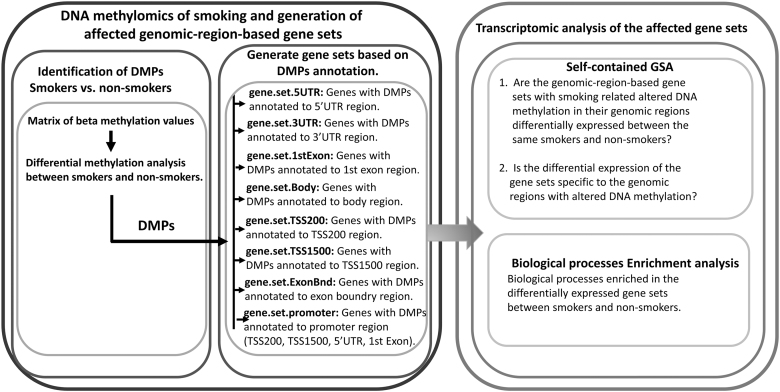
Schematic diagram representing the overall gene set based integrative multi-omics analysis for studying the impact of cigarette smoking on DNA methylation and its transcriptomic consequences. DMPs, differentially methylated positions; GSA, gene set analysis.

Integrative analysis of DNA methylation and transcriptomics data concerning the effects of smoking habit was done by conducting self-contained gene set analysis of the differentially methylated gene sets using rotation gene set test (*ROAST*) (Wu et al., 2010) implemented in *limma* R/Bioconductor package (Fig. 2).

## Results

### Study participants

The characteristics of the YFS cohort participants of this study are shown in Table 1. Only 150 out of the total 1114 participants were daily smokers. The proportion of male participants was higher among daily smokers (53%) as compared with the non-smokers group (45%). Daily smokers were less involved in physical activity and had higher alcohol consumption as compared with never or less than daily smokers.

**Table 1.** Population Characteristics of the Young Finns Study Participants

Characteristics	Daily smokers	Never or less than daily smokers
Number of subjects	150	964
Sex (% women and % men)	47% and 53%	55% and 45%
Age, years	41 ± 5 (Range 34–49)	42 ± 5 (Range 34–49)
Body mass index, kg/m^2^	26.1 ± 4.1	26.6 ± 5.1
Total cholesterol (mmol/L)	5.0 ± 0.9	5.0 ± 0.9
Low-density lipoprotein (LDL) cholesterol (mmol/L)	3.0 ± 0.8	3.1 ± 0.8
High-density lipoprotein (HDL) cholesterol (mmol/L)	1.3 ± 0.3	1.3 ± 0.3
Systolic blood pressure (mmHg)	120.4 ± 12.7	120.4 ± 14.4
Diastolic blood pressure (mmHg)	73.9 ± 10.7	75.8 ± 11.4
Alcohol consumption, units/day	1.4 ± 1.6	0.7 ± 1.1
Physical activity index (MET h/week)	12.3 ± 15.5	20.6 ± 22.2
Socioeconomic status	Low: 29%	Low: 18%
	Medium: 43%	Medium: 39%
	High: 28%	High: 43%

Data are mean ± SD or proportions.

MET-h, metabolic equivalent task hour; SD, standard deviation.

### DMPs between active smokers and never smokers

**FIG. 3.**
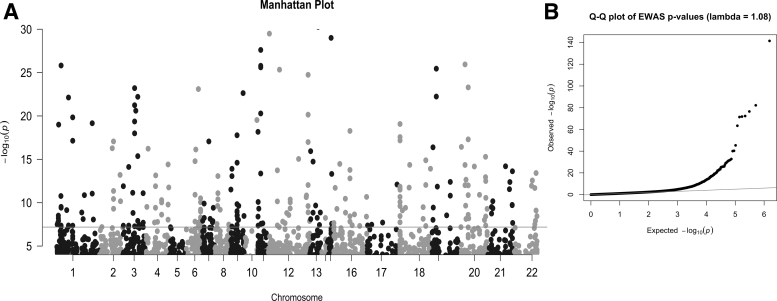
(**A**) Manhattan plot showing the *p*-values of genome-wide CpG sites. *X*-axis represents the position of the CpG sites on each chromosome. *Y*-axis represents the negative log10 of the *p*-values for the association. The *solid horizontal line* represents the genome-wide significance threshold (*p* = 6.5 × 10^−8^). (**B**) Quantile-quantile plot showing genomic inflation factor (*λ* = 1.08) of the epigenome-wide association study. The genomic inflation factor (ratio of the median of the empirically observed distribution of the test statistic to the expected median) represents the extent of inflation and false positive rate in the results.

In EWAS comparing active smokers (*N* = 150) and never or less than daily smokers (*N* = 964), we identified 272 statistically significant CpG sites or DMPs with methylome-wide significance (*p* < 6.5 × 10^−8^) and 1206 DMPs with a more liberal threshold of FDR <0.05 (Fig. 3). The genomic inflation factor (*λ*) for these results was 1.08, which is lower than that reported in other studies (Christiansen et al., 2021; Joehanes et al., 2016). The 272 statistically significant DMPs in our results constituted 36 novel smoking-related DMPs (Table 2) and replicated 236 DMPs from previous studies (Christiansen et al., 2021; Joehanes et al., 2016) (Supplementary Table S1). The number of DMPs replicated in our study based on FDR <0.05 was 663.

**Table 2.** List of 36 Novel Cigarette Smoking-Related CpG Sites Identified in This Study with Genome-Wide Significance of *p*-Value <6.5 × 10^−^^8^ (0.05/769,683), Their Corresponding Genomic Location, Corresponding Genes, Coefficients, Standard Error, and *p*-Values

ProbeID	Chromosome	Position	Genes	Coefficients	SE	*p*
cg12637027	chr5	56690874		−0.026	0.003	4.4 × 10^−15^
cg14486033	chr2	54643636		−0.018	0.002	1.9 × 10^−14^
cg19136686	chr16	17464401	*XYLT1*	−0.009	0.001	8.6 × 10^−14^
cg00592949	chr9	112680911	*PALM2*; *PALM2-AKAP2*	−0.032	0.004	2.7 × 10^−13^
cg12739216	chr12	131706350		−0.025	0.004	1.7 × 10^−12^
cg15775568	chr2	54643284		−0.013	0.002	7.1 × 10^−11^
cg24947681	chr15	39760933		−0.021	0.003	1.3 × 10^−10^
cg16485845	chr8	141802466	*PTK2*	−0.014	0.002	1.8 × 10^−10^
cg24087280	chr17	48193712	*SAMD14*	0.015	0.002	2.3 × 10^−10^
cg15548246	chr6	13121401	*PHACTR1*	0.013	0.002	3.6 × 10^−10^
cg02511321	chr5	32098574	*PDZD2*	0.015	0.002	3.9 × 10^−10^
cg10682119	chr15	93182494	*FAM174B*	−0.034	0.005	3.9 × 10^−10^
cg22311669	chr16	30466567		−0.016	0.003	6.4 × 10^−10^
cg06563667	chr1	58016025	*DAB1*	−0.019	0.003	7.2 × 10^−10^
cg04481318	chr9	134282053		−0.022	0.004	8 × 10^−10^
cg26823705	chr1	145435523	*NBPF20*; *NBPF10*	−0.024	0.004	1.3 × 10^−9^
cg15102575	chr3	124510809	*ITGB5*	−0.021	0.003	2.2 × 10^−9^
cg11075883	chr7	146658441	*CNTNAP2*	0.013	0.002	3.9 × 10^−9^
cg07815896	chr15	40385132	*BMF*	−0.017	0.003	5.7 × 10^−9^
cg26894575	chr1	153518054	*S100A4*	−0.006	0.001	7.8 × 10^−9^
cg13518852	chr1	212892006		0.026	0.005	8.7 × 10^−9^
cg18734657	chr7	139420591	*HIPK2*	−0.023	0.004	1 × 10^−8^
cg00442581	chr9	130733834	*FAM102A*	−0.022	0.004	1.2 × 10^−8^
cg08151621	chr19	28995456	*LOC100420587*	−0.017	0.003	2.4 × 10^−8^
cg01990910	chr16	12207648	*SNX29*	−0.016	0.003	2.9 × 10^−8^
cg06467473	chr9	127054510	*NEK6*	−0.011	0.002	3.1 × 10^−8^
cg19467605	chr11	94349883	*PIWIL4*	0.030	0.005	3.4 × 10^−8^
cg07411532	chr20	56266785	*PMEPA1*	0.013	0.002	3.7 × 10^−8^
cg09465516	chr2	54751985	*SPTBN1*	−0.021	0.004	4.2 × 10^−8^
cg16762439	chr7	146795245	*LOC101928700*; *CNTNAP2*	0.013	0.002	4.3 × 10^−8^
cg20430809	chr19	2089006	*MOB3A*	−0.014	0.003	4.4 × 10^−8^
cg05702597	chr3	154829011	*MME*	−0.025	0.004	4.7 × 10^−8^
cg11786988	chr17	8826387	*PIK3R5*	−0.012	0.002	5.6 × 10^−8^
cg21811986	chr3	5053132		−0.012	0.002	6 × 10^−8^
cg17340043	chr10	13202710	*MCM10*	0.021	0.004	6.4 × 10^−8^
cg11674355	chr2	65610261	*SPRED2*	−0.024	0.004	6.4 × 10^−8^

Full list of smoking-related CpG sites identified in this study can be found in Supplementary Table S1.

SE, standard error.

### DEGs between active smokers and never smokers

**FIG. 4.**
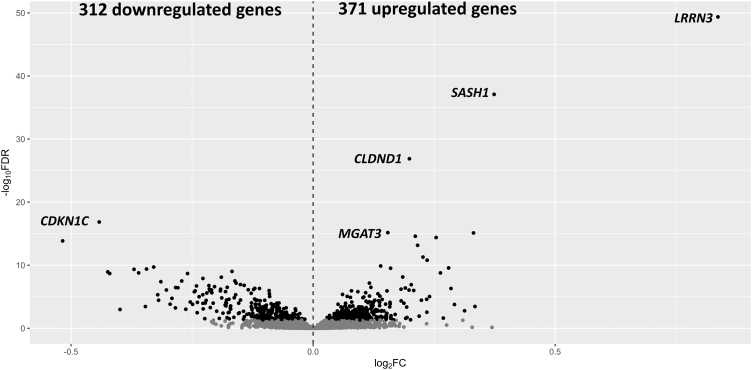
Volcano plot of differentially expressed genes between active smokers and non-smokers. The dots on the right and left sides of the dashed vertical line represent 371 up and 312 downregulated genes, respectively, among active smokers with FDR <0.05. The *grey dots* represent statistically not significant genes with FDR >0.05. The five most significant differentially expressed genes are labeled. The *y*-axis represents negative log (base10) of FDR and the *x*-axis represents log (base 2) of the fold change between active smokers and non-smokers. FDR, false discovery rate.

Differential gene expression (DGE) analysis of transcriptomics data identified 371 genes upregulated and 312 genes downregulated (683 DEGs) among daily smoking participants with FDR <0.05 (Fig. 4) (Supplementary Table S2). While GO based gene set enrichment analysis of the 371 upregulated genes identified 105 biological processes with FDR <0.05 (Supplementary Table S3), only 87 biological processes were identified to be enriched in the 312 downregulated genes with FDR <0.05 (Supplementary Table S4).

Majority of the significantly enriched biological process GO terms in the upregulated genes were related to immune system, metabolism of carbohydrate, metabolism of fat, and signaling cascade. Similarly, GO terms enriched in the downregulated genes included biological processes such as regulation of cell killing, leukocyte-mediated cytotoxicity, lymphocyte proliferation, T cell activation, T cell receptor signaling pathway, immunological memory process, heart trabecula formation, immune response to tumor cell, pyroptosis, and interferon-gamma production.

Among the 683 smoking-related DEGs identified in this study, only 11 genes (6 up- and 5 downregulated) had altered methylation level in their genomic regions. While three of the six upregulated genes (leucine rich repeat neuronal 3 [*LRRN3*], *MGAT3* and G protein-coupled receptor 15 [*GPR15*]) had hypomethylated CpGs in their promoter region, the other three (claudin domain-containing protein 1 [*CLDND1*], *FAM102A*, and *EPHA4*) had hypomethylation in their body region.

Among the five downregulated genes, two (*PRSS23* and *SLAMF7*) had hypomethylation in their promoter region and the other three (*MTSS1*, *GFI1*, and *CCM2*) had hypomethylation in their body region.

### Association between transcriptomic level gene expression and smoking-related alterations in DNA methylation

The gene set containing 49 genes with hypomethylated CpG sites in their body region was differentially expressed among smokers for both mixed hypothesis (the genes are up- or downregulated) and up hypothesis (the genes are upregulated) with FDR.mixed = 0.0005 and FDR.up = 0.03, respectively. The three most statistically significant upregulated genes (based on DGE analysis) in the gene set were *SIN3B* (FDR = 0.06), *BMF* (FDR = 0.08), and *PDE1C* (FDR = 0.19).

**FIG. 5.**
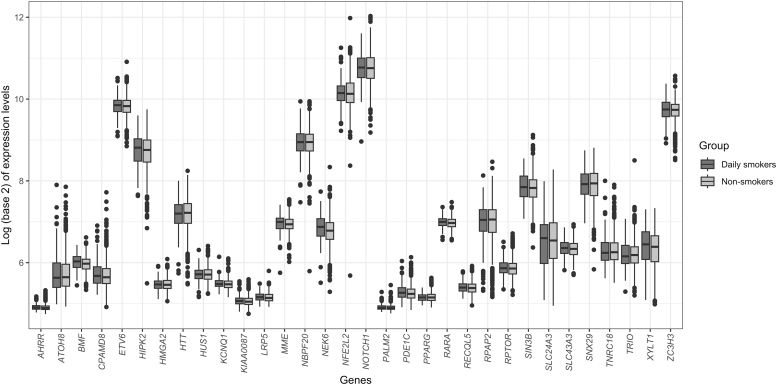
Box plots for gene expression changes for the 32 upregulated genes with hypomethylated CpG sites in their body region between daily smokers and never or less than daily smokers.

**FIG. 6.**
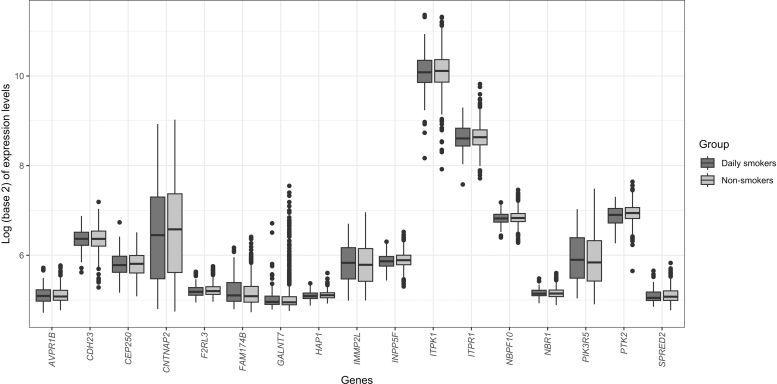
Box plots for gene expression changes for the 17 downregulated genes with hypomethylated CpG sites in their body region between daily smokers and never or less than daily smokers.

Difference in expression levels of all the 32 upregulated genes in this gene set is shown in Figure 5. Similarly, the three most DGE analysis-based downregulated genes in the gene set were *ITPK1* (FDR = 0.49), *CDH23* (FDR = 0.49), and *NBR1* (FDR = 0.59). Difference in expression levels of all the 17 downregulated genes in this gene set is shown in Figure 6. Overall, genes in the gene set were enriched in GO terms related to biological processes such as ossification, metal ion transport, response to oxygen levels, fat cell differentiation, cell death, and peptidyl-serine phosphorylation (Supplementary Table S5).

**FIG. 7.**
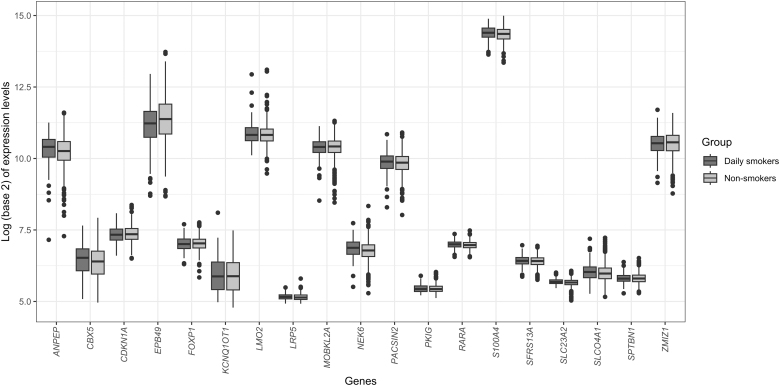
Box plots for gene expression changes for the 19 upregulated genes with hypomethylated CpG sites in their promoter region between daily smokers and never or less than daily smokers.

The other gene set containing 33 genes with hypomethylated CpG sites in their promoter region was differentially expressed among smokers only for mixed hypothesis with FDR.mixed = 0.001. The three most DGE analysis-based statistically significant upregulated genes in the gene set were *SLC23A2* (FDR = 0.09), *ANPEP* (FDR = 0.16), and *NEC6* (FDR = 0.28). Difference in expression levels of all the 19 upregulated genes in this gene set is shown in Figure 7.

**FIG. 8.**
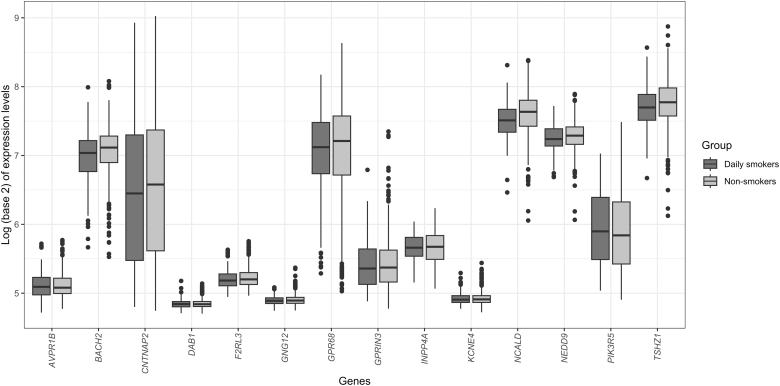
Box plots for gene expression changes for the 14 downregulated genes with hypomethylated CpG sites in their promoter region between daily smokers and never or less than daily smokers.

The three most downregulated genes in the gene set were *NCALD* (FDR = 0.07), *INPP4A* (FDR = 0.21), and *BACH2* (FDR = 0.25). Difference in expression levels of all the 14 downregulated genes in this gene set is shown in Figure 8. Member genes of this gene set were enriched in the biological process GO term related to the cerebral cortex development process.

None of the gene sets containing genes with hypermethylated genomic positions were differentially expressed among smokers with a statistical significance threshold of FDR <0.05.

## Discussion

In the present study, we performed a novel advanced gene set based integrative analysis of DNA methylomic and transcriptomic data to identify epigenetic alterations associated with smoking and its transcriptomic consequences. The integrative system-level approach allowed us to identify sets of novel genes that have smoking-related alterations in DNA methylation within their genomic regions and are also differentially expressed among smokers.

The novelty of this study rests in the usage of both a novel data platform and a novel advanced system-level statistical method to study epigenetic and transcriptomic consequences of smoking. To our knowledge, this is the first multi-omics integrative study of smoking that is based on EPIC BeadChip-based DNA methylation and transcriptomic data from whole blood.

The number of measured CpG sites in EPIC BeadChip is nearly twice as many the number in HumanMethylation450 BeadChip (450 K), generating an important scientific gap that needs to be addressed. On the statistical method side, we propose a novel system-level integrative approach that pools and analyzes together all the genes based on their genomic regions where smoking-related DNA methylation alterations have occurred. Our results showed that this approach is statistically more powerful as compared with traditional one-to-one association analysis between genes and methylation sites such as the study by Maas et al. (2020).

Gene sets containing genes with hypomethylated CpG sites in body as well as in promoter regions were significantly differentially expressed among smokers. While the gene set containing genes with hypomethylated CpG sites in their body region was upregulated among smokers, the one with hypomethylated CpG sites in the promoter region contained both up- and downregulated genes among smokers. Interestingly, genes from the gene sets based on both promoter and body regions were not found to be differentially expressed using the traditional gene-wise differential gene expression analysis in this study.

Similarly, to the best of our knowledge, the effects of smoking-related differentially methylated genes on gene expression have not been identified in any of the previous transcriptome-wide studies despite the pronounced association of smoking with DNA methylation in the genomic regions of some of the member genes such as *AHRR* and *F2RL2* reported by several studies such as Joehanes et al. (2016), Kaur et al. (2019), and Zeilinger et al. (2013).

The novel genes within the gene sets identified in this study are enriched in biological processes that are known in literature to have associations with smoking; however, the underlying molecular mechanisms remain mostly unexplained. This study revealed the epigenetic mechanisms through which these biological processes are affected by smoking. For example, smoking is a well-known risk factor for osteoporosis (Yoon et al., 2012); this study identified the smoking-triggered epigenetic mechanism that affects genes involved in the process of bone formation.

Similarly, this study identified that genes involved in metal ion transport are affected by smoking-related alterations in DNA methylation. Imbalance in metal ion concentration in cerebrospinal fluid caused by smoking can lead to cognitive impairment (Li et al., 2021). Similarly, alterations in metal ion transport system can affect copper homeostasis, which, in turn, can lead to dyslipidemia and low-density lipoprotein oxidation (Meyer et al., 2014) and its related diseases such as osteoporosis (Poiana et al., 2013) and atherosclerosis (Linton et al., 2019).

Despite being one of the most important and preventable risk factors for atherosclerosis, the underlying molecular mechanism linking smoking and atherosclerosis remains largely unknown. Our findings support the hypothesis that the atherogenic effect of smoking might involve endothelial cell death (Messner et al., 2012). We identified that genes involved in cerebral cortex development are differentially expressed among smokers through the epigenetic mechanism. Our finding supports a previous study that reported an association between smoking and accelerated cortical thinning that causes cognitive decline in adults (Karama et al., 2015).

Univariate analysis of DNA methylation and transcriptomic data separately concerning smoking also generated novel results in this study. Biological implications of the novel DMPs along with the replicated ones from DNA methylation data analysis were studied with the downstream integrative approach as described earlier.

The largest transcriptomic study of smoking so far by Huan et al. (2016) reported 1270 DEGs between active smokers and never smokers at FDR <0.1, out of which 289 were replicated in this study at the same statistical significance threshold (FDR <0.1). Similarly, we also replicated 69 smoking-related genes reported by another study (Vink et al., 2017).

Considering the smoking-related genes reported in both the studies (Huan et al., 2016; Vink et al., 2017), there were 428 new genes at FDR <0.05 and 694 new genes at FDR <0.1 reported in this study. The most significant smoking-related genes identified in this study such as *LRRN3*, *CLDND1*, *PID1* (phosphotyrosine interaction domain-containing protein 1), *GPR15*, and *S1PR5* (sphingosine-1 phosphate receptor 5) were consistent in all other recent studies reviewed here.

Biological processes linked to the DEGs from transcriptomic data analysis such as immune system, cell death, fat metabolism, and signaling cascade are known to play a central role in the development of atherosclerosis (Hultén and Levin, 2009; Kong et al., 2022; Messner et al., 2012; Wolf and Ley, 2019).

This study has several limitations, one of them being the self-reported smoking status. However, this study replicated the most consistent findings across the literature from both DNA methylation and transcriptomics data indicating the robustness of the data and analysis approach. Another limitation is that the study was based on Infinium MethylationEPIC (EPIC) BeadChips for DNA methylation and Illumina HumanHT-12 version 4 Expression BeadChip for transcriptome, which provide suboptimal coverage of regulatory elements as compared with sequencing-based platforms such as whole-genome bisulfite sequencing and RNA-Seq.

The study was based on cross-sectional data, and, therefore, changes in DNA methylation after cessation of smoking could not be studied. Also, the participants of this study are of European origin and therefore further studies with populations of different ethnicities are needed. However, this study contributed a novel gene-set based integrative DNA methylome and transcriptome analysis approach. The approach is statistically more powerful as compared with the traditional single-molecule analysis methods and can allow identification of small but coordinated changes in gene expression associated with DNA methylation.

## Conclusions

Using system-level integrated analysis of DNA methylation and transcriptomics data, we identified novel sets of genes associated with smoking through epigenetic alterations, uncovering the potential molecular cascade underlying the disease induction mechanism by smoking. The genes and their epigenetic regulation explain the underlining mechanism of how smoking affects different biological processes such as those related to the immune system, metal ion transport, osteoblast differentiation, hypoxia, cell death, and the cerebral cortex development process and can lead to related diseases such as atherosclerosis, osteoporosis, and cognitive impairment.

Importantly, this study proposes an alternative system-level integrative multi-omics analysis method that can uncover small but coordinated changes in gene expression potentially due to DNA methylation changes in their genomic regions. Such small and coordinated changes in gene expression can be missed by traditional linear association analysis methods due to lack of sufficient statistical power. The proposed integrative method can be applied to study a wide range of biological problems, for example, to uncover epigenetic alterations associated to a disease or a trait of interest, their effects on gene expression and related biological processes.

## Supplementary Material

Supplemental data

Supplemental data

Supplemental data

Supplemental data

Supplemental data

## Abbreviations Used

BMI: body mass index
*CLDND1*: claudin domain-containing protein 1
DEG: differentially expressed gene
DMP: differentially methylated position
DNMT1: DNA methyltransferase 1
EWAS: epigenome-wide association study
FDR: false discovery rate
GO: gene ontology
*GPR15*: G protein-coupled receptor 15
GSA: gene set analysis
*LRRN3*: leukine-rich repeat neuronal 3
MET-h: metabolic equivalent task hours
OR: odds ratio
SD: standard deviation
SE: standard error
TSS: transcription start site
UTR: untranslated region
YFS: Young Finns Study
